# Optimised Neutralisation Strategies for Validating the Virucidal Efficacy of Micro-Chem Plus™ Against High-Containment Negative-Sense RNA Viruses

**DOI:** 10.3390/v17111424

**Published:** 2025-10-27

**Authors:** Xiaoxiao Gao, Cheng Peng, Chao Shan, Yanfeng Yao, Zhiming Yuan

**Affiliations:** 1 State Key Laboratory of Virology and Biosafety, Wuhan Institute of Virology, Chinese Academy of Sciences, Wuhan 430071, China; gaoxiaoxiao@wh.iov.cn (X.G.); pengcheng@wh.iov.cn (C.P.); shanchao@wh.iov.cn (C.S.); 2 Center for Biosafety Mega-Science, Wuhan Institute of Virology, Chinese Academy of Sciences, Wuhan 430071, China; 3 University of the Chinese Academy of Sciences, Beijing 100049, China

**Keywords:** Micro-Chem Plus™ (MCP), disinfectant validation, MCP neutralisation, biosafety, BSL-4

## Abstract

Effective disinfectant validation is essential for ensuring biosafety in high-containment laboratories when lethal pathogens are being handled. Micro-Chem Plus™ (MCP) is widely used in high-containment facilities for pathogen disinfection and routine decontamination. However, it induces severe cytotoxicity in cell culture, which may lead to an overestimation of its virucidal efficacy during disinfectant validation assays. To resolve this problem, we systematically evaluated the effects of three neutralisation methods (dilution, chemical neutralisation, and chromatographic separation) on MCP. The results showed that a 400-fold dilution with assay medium completely neutralised MCP, but reliable detection required high viral titers (≥6 log_10_ TCID_50_/mL). Chemical neutralisation using Dey–Engley broth showed inherent cytotoxicity, while chromatographic separation (MicroSpin S-400 HR/DetergentOUT™ columns) was the most effective but necessitated an additional 8-fold dilution. Validation in a BSL-4 facility with the risk group 4 (RG-4) agent Ebola virus confirmed MCP’s concentration- and time-dependent virucidal activity, achieving a ≥6 log_10_ TCID_50_ reduction within 1–5 min. This study establishes an optimised framework for disinfectant validation in high-containment laboratories, addressing critical gaps in current protocols.

## 1. Introduction

Micro-Chem Plus™ (MCP), a dual quaternary ammonium compound (QAC)-based disinfectant, has been widely adopted in high-containment laboratories (Biosafety Levels 3 and 4, BSL-3/4) due to its material compatibility with chemical shower systems and proven efficacy against enveloped viruses [1,2,3,4,5,6,7]. Before implementation in BSL-4 facilities, disinfectants must undergo stringent validation to ensure reliable inactivation of high-risk pathogens. Current validation workflows face two major limitations: (i) the absence of standardised neutralisation protocols for dual-QAC formulations, where conventional chemical neutralisers (e.g., Dey–Engley broth) may exhibit cytotoxicity or produce assay-interfering byproducts, and (ii) cumbersome, expertise-dependent methods that impose impractical burdens on maximum-containment operations [8].

Neutralisation in disinfectant validation serves a dual purpose: quenching residual antimicrobial activity to prevent false-negatives and mitigating cytotoxicity to enable accurate viability assessment. While dilution, chemical neutralisation, and column-based techniques are widely employed, their comparative performance for dual-QAC formulations remains uncharacterised—a critical gap given the increasing use of MCP in high-containment settings [3,9,10,11,12,13,14,15,16,17]. To address this, we systematically evaluated these neutralisation strategies using vesicular stomatitis virus (VSV), a BSL-2 surrogate for enveloped viruses, and validated their operational applicability under BSL-4 conditions with Ebola virus (EBOV) [18].

By integrating mechanistic validation with translational biosafety practices, this work establishes a standardised, resource-efficient framework for disinfectant testing in high-containment laboratories. Our data provide actionable protocols for regulatory compliance and risk mitigation, advancing the paradigm of virucidal efficacy testing for high-risk pathogens.

## 2. Materials and Methods

### 2.1. Cell Lines and Viruses

Vero E6 cells (African green monkey kidney epithelial cells), obtained from the National Virus Resource Center, Wuhan Institute of Virology, Chinese Academy of Sciences, were maintained in Dulbecco’s Modified Eagle Medium (DMEM, Gibco, Thermo Fisher Scientific, Waltham, MA, USA) supplemented with 10% foetal bovine serum (FBS, Gibco, Thermo Fisher Scientific, Waltham, MA, USA). The cells were cultivated under humidified conditions with 5% CO_2_ at 37 °C.

Recombinant vesicular stomatitis virus expressing green fluorescent protein (VSV-GFP) was kindly provided by Prof. Rongjuan Pei. Ebola virus (EBOV, Mayinga 1976 strain) was obtained from the National Virus Resource Center, Wuhan Institute of Virology, Chinese Academy of Sciences. All experiments containing EBOV were performed in biosafety level 4 (BSL-4) facilities of the National Biosafety Laboratory, Wuhan, Chinese Academy of Sciences, following approved standard operating procedures. The viruses were propagated in Vero E6 cells at a multiplicity of infection (MOI) of 0.01. All virus stocks were titrated using a 50% tissue culture infectious dose (TCID_50_) assay, aliquoted, and stored at −80 °C until use.

### 2.2. Suspension Test

Micro-Chem Plus™ (MCP; National Chemical Laboratories, Inc., Philadelphia, PA, USA) was freshly diluted to the corresponding concentrations (*v*/*v*) with soft water. Equal volumes (100 μL) of virus stock and disinfectant were mixed and incubated at 22 ± 2 °C for predetermined contact times (1–5 min).

### 2.3. Neutralisation Methods

Three independent neutralisation strategies were evaluated to terminate MCP activity while preserving potential surviving virions.

#### 2.3.1. Dilution-Based Neutralisation

Virus–disinfectant mixtures were immediately diluted in DMEM supplemented with 2% FBS (assay medium). This physical quenching method was selected to rapidly reduce the MCP concentration below the virucidal threshold.

#### 2.3.2. Chemical Neutralisation

Samples were treated with Dey–Engley (D/E) neutralising broth (BD Difco™ and Huankai) at a 1:19 (*v*/*v*) ratio, achieved by mixing one volume of the virus–MCP mixture with nineteen volumes of the D/E neutralising broth. In a typical experiment, 50 µL of the virus–MCP mixture was added to 950 µL of D/E broth. Following 15 min of incubation at 22 ± 2 °C, the neutralised samples were subjected to secondary dilution to eliminate residual cytotoxicity from the neutraliser components.

#### 2.3.3. Chromatographic Separation

Two column-based chromatographic methods were employed. Size-exclusion chromatography was carried out with MicroSpin™ S-400 HR columns (Cytiva, Marlborough, MA, USA), and detergent removal was performed using DetergentOUT™ GBS10-800 columns (G-Biosciences, St. Louis, MO, USA). All columns were pre-equilibrated with sterile water prior to sample loading. After applying the samples, centrifugation was performed at 700× *g* for 1 min. The collected eluates were immediately diluted in assay medium to eliminate any residual disinfectant activity.

### 2.4. Cytotoxicity Assay

Cell viability was quantified using a Cell Counting Kit-8 (CCK-8; Vazyme Biotech Co., Ltd., Nanjing, China) according to the manufacturer’s specifications, and the absorbance was measured at 450 nm using a Tecan Infinite^®^ 200 PRO microplate reader. For each treatment, three biological replicates (*n* = 3) were analysed. The percentage of viable cells was calculated as follows: % viability = [(OD_treatment_ − OD_blank_)/(OD_control_ − OD_blank_)] × 100, where OD_treatment_ = mean absorbance of treated cells; OD_blank_ = mean absorbance of reagent blank (CCK-8 in medium without cells); and OD_control_ = mean absorbance of untreated control cells.

### 2.5. Viral Titration

Serial 10-fold dilutions of neutralised samples were inoculated onto Vero E6 monolayers in 96-well plates (*n* = 6 wells/dilution). After 10 days of incubation, cytopathic effects were examined microscopically. The TCID_50_ values were calculated using the Reed–Muench method.

### 2.6. Viral Inactivation Validation

Viral inactivation was assessed through three sequential blind passages in Vero E6 cells, during which at least half of the culture supernatant from each passage was used to inoculate fresh cells. This was followed by quantitative evaluation using probe-based qRT-PCR (HiScript^®^ II One-Step qRT-PCR Probe Kit, Vazyme Biotech). The following oligonucleotides were employed: forward primer 5′-ATTTGAATGGGGTCCAATTGCC-3′ [EBOV-L-Q(F)], reverse primer 5′-AAGCAGTRCCTATACTAGCCA-3′ [EBOV-L-Q(R)], and probe 5′-FAM-AGTCCCTTAAAACGGCTACAAGAATGGGAC-BHQ1-3′ [EBOV-L-Q(P)] [19]. Complete inactivation was defined by the absence of cytopathic effects and undetectable genomic RNA (<10 copies/μL) in the third passage.

### 2.7. Experimental Design

Cytotoxicity was evaluated using a tiered approach to assess both individual components and combined treatments, as detailed in Table 1. Following 3 h of treatment, cell viability was assessed using a previously described method.

To systematically evaluate the virucidal activity of MCP and the effectiveness of the neutralisation methods, we designed six experimental groups (Table 2). After the respective treatments, the VSV titers were quantified by a TCID_50_ assay.

### 2.8. Statistical Analysis

Statistical tests were performed using GraphPad Prism 5. Data are expressed as the means ± standard deviations (SDs).

## 3. Results

### 3.1. Dilution-Based Neutralisation Achieves Complete MCP Inactivation

**Figure 1:**
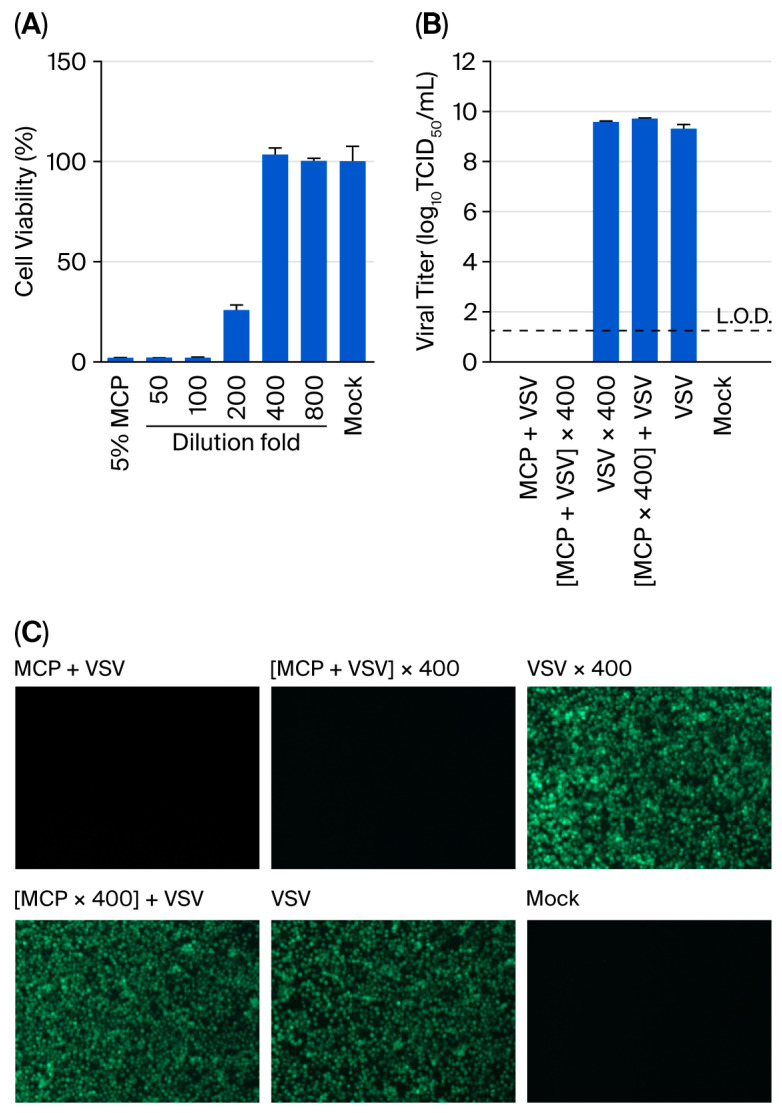
Dilution-based neutralisation of MCP demonstrates concentration-dependent efficacy. (**A**) Cytotoxicity assessment of MCP dilutions (*n* = 3). Vero E6 cells were incubated with serially diluted 5% MCP in DMEM supplemented with 2% FBS for 3 h, followed by cell viability measurement using a CCK-8 assay. (**B**) Viral infectivity after neutralisation treatment (*n* = 3). Viral titers were determined using a TCID_50_ assay following the indicated treatments. Experimental conditions: (I) virus treated with 5% MCP for 5 min, (II) MCP-treated virus (5 min) followed by a 400-fold dilution, (III) virus subjected to a 400-fold dilution alone, (IV) virus incubated with 400-fold diluted 5% MCP, (V) untreated virus control, (VI) assay medium. Viral titers were calculated based on the initial viral load prior to dilution. Data represent the means ± SDs; the dashed line indicates the limit of detection (L.O.D., 1.25 log_10_ TCID_50_/mL). (**C**) Fluorescence microscopy validation of neutralisation efficacy.

The neutralisation capacity of serial dilutions in DMEM supplemented with 2% FBS was systematically evaluated against the standard 5% MCP formulation following a 5 min contact time. Cytotoxicity assessment revealed a concentration-dependent response, with 400-fold dilution established as the minimum requirement for complete neutralisation (Figure 1A). Undiluted 5% MCP reduced Vero E6 cell viability to 2.1 ± 0.1%, while intermediate 200-fold dilution showed partial cytotoxicity (25.9% viability). Complete restoration of cellular viability (103.4 ± 4.9%) was achieved at 400-fold dilution. Morphological assessment through microscopic examination confirmed these findings, demonstrating the absence of cytotoxicity-related changes (cell rounding, shrinkage, detachment, and lysis) at this dilution threshold.

The subsequent neutralisation efficacy validation employed a multimodal assessment strategy combining fluorescence microscopy with TCID_50_ titration. All viral titers were normalised to the same initial viral load. This approach yielded several critical findings (Figure 1B,C): (i) 5% MCP treatment alone resulted in complete inactivation of VSV (9.3 ± 0.2 log_10_ TCID_50_/mL reduction), with no detectable fluorescence or replicative virus posttreatment; (ii) 400-fold diluted virus–disinfectant mixtures showed no evidence of infectivity restoration, confirming effective neutralisation; (iii) the dilution process itself had no measurable impact on viral infectivity (9.5 ± 0.1 log_10_ TCID_50_/mL); and (iv) neutralised MCP solutions exhibited no intrinsic antiviral activity. The control groups confirmed the validity of the assay, establishing a 400-fold dilution as a reliable neutralisation method for suspension studies.

### 3.2. Chemical Neutralisation Reveals Formulation-Specific Limitations

Dey–Engley (D/E) broth is widely employed for neutralising conventional QACs, and the efficacy of two commercial preparations (BD Difco™ and Huankai) have been assessed for their efficacy in neutralising MCP.

**Figure 2:**
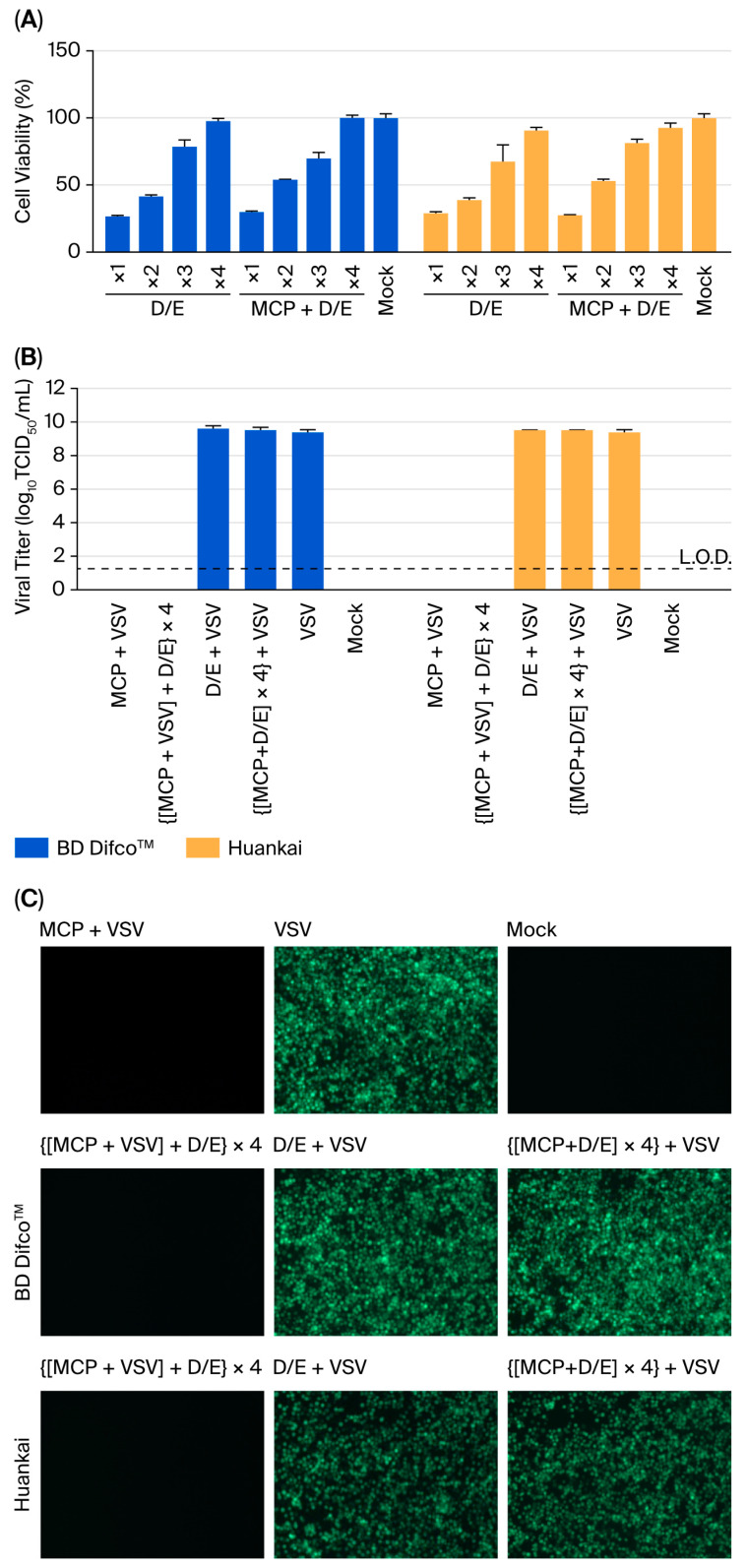
Chemical neutralisation with D/E broth reveals formulation-specific limitations. (**A**) Cytotoxicity assessment of neutralisation components (*n* = 3). Cell viability was measured by a CCK-8 assay after 3 h of exposure to (I) serially diluted (1–4-fold) D/E neutralising solution, (II) neutralised MCP-D/E mixture (1–4-fold diluted), and (III) assay medium. (**B**) Viral infectivity following various treatments (*n* = 3). VSV titers were quantified using the TCID_50_ assay under the following conditions: (I) MCP treatment alone, (II) D/E neutralisation post-MCP treatment (followed by 4-fold dilution), (III) D/E solution alone, (IV) MCP and D/E co-treatment, (V) untreated virus control, (VI) culture medium control. Viral titers were calculated based on the initial viral load prior to neutralisation. The bars show the means ± SDs; L.O.D. = 1.25 log_10_ TCID_50_/mL. (**C**) Fluorescence microscopy across treatment conditions.

Initial cytotoxicity screening revealed that both D/E preparations exhibited intrinsic cytotoxicity, reducing Vero E6 cell viability to 26.5 ± 1.2% (BD Difco™) or 28.8 ± 1.8% (Huankai) after 3 h of exposure (Figure 2A). The cytotoxic effect was successfully eliminated through a 4-fold dilution with DMEM supplemented with 2% FBS, which was subsequently applied to the following chemical neutralisation experiments. The optimised neutralisation protocol involved (i) 1:19 (*v*/*v*) mixing of 5% MCP with D/E broth, (ii) a 15 min incubation at room temperature (22 ± 2 °C), and (iii) a 4-fold secondary dilution. This protocol completely abolished the cytotoxic effects of MCP while maintaining system integrity for subsequent assays.

Validation studies using this protocol were performed with both fluorescence microscopy and TCID_50_ assays. As illustrated in Figure 2B,C, the following key results were consistently observed: (i) 5% MCP treatment alone resulted in complete inactivation of VSV, with no detectable infectivity remaining after treatment; (ii) no restoration of viral infectivity was observed following D/E neutralisation after MCP treatment; (iii) D/E solution alone showed no adverse effects on viral infectivity; (iv) neutralised MCP mixtures resulted in no residual antiviral activity; and (v) all system controls performed within the expected parameters. Importantly, the neutralisation process did not interfere with the viral detection systems, confirming the specificity and reliability of these observations. Together, these findings indicate that the dual-QAC formulation of MCP likely requires alternative neutralisation strategies that extend beyond conventional D/E broth.

### 3.3. Chromatographic Methods Require Supplemental Dilution

Two column-based systems were systematically compared: size-exclusion chromatography (MicroSpin S-400 HR) and detergent-removal columns (DetergentOUT™ GBS10-800). Initial cytotoxicity assessment demonstrated that while both columns effectively reduced the MCP concentration, the eluates still retained significant cytotoxicity.

**Figure 3:**
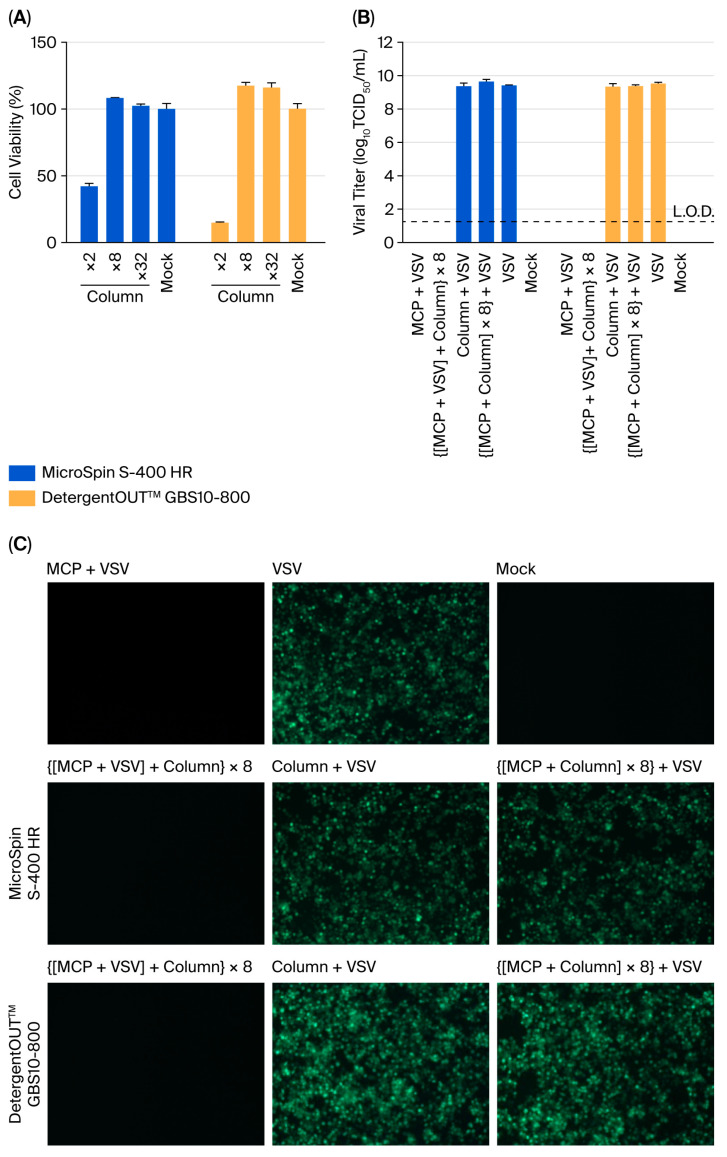
Chromatographic methods requires supplemental dilution for complete MCP neutralisation. (**A**) Cytotoxicity assessment of column-processed MCP (*n* = 3). The eluate from 5% MCP treated with columns was diluted 2-, 8-, or 32-fold in DMEM supplemented with 2% FBS and incubated with Vero E6 cells for 3 h. Cell viability was measured by a CCK-8 assay. (**B**) Viral titer determination by TCID_50_ assay (*n* = 3). Treatment conditions: (I) 5% MCP alone (5 min of exposure), (II) column-based processing of MCP-inactivated virus (8-fold diluted eluate), (III) column-processed virus control, (IV) 8-fold diluted MCP eluate incubated with native virus, (V) untreated virus, (VI) assay medium. Viral titers were calculated based on the initial viral load prior to column treatment. The values represent the means ± SDs; the dashed line indicates the L.O.D. (1.25 log_10_ TCID_50_/mL). (**C**) Fluorescence microscopy after the indicated treatments.

To eliminate the cytotoxicity of the eluate, it was diluted with assay medium after filtration. Take MicroSpin S-400 HR column as an example (Figure 3A). Quantitative analysis of postcolumn dilution effects revealed that (i) 2-fold diluted eluates maintained substantial cytotoxicity (41.9 ± 3.4% viability), (ii) 8-fold dilution achieved complete noncytotoxicity (108.2 ± 0.5% viability), and (iii) 32-fold dilution provided no additional benefit (102.1 ± 2.3% viability).

Based on these findings, an 8-fold dilution with assay medium was established for all subsequent neutralisation efficacy studies. Validation experiments demonstrated that (i) the 5% MCP solution effectively inactivated VSV; (ii) following treatment with either column to remove residual disinfectant, VSV failed to regain cellular infectivity; (iii) neither column exhibited any detectable effect on VSV infectivity; and (iv) the 8-fold diluted eluate showed neither inhibitory effects on VSV infection nor interference with the detection assay (Figure 3B,C).

Notably, both columns showed comparable performance, with complete neutralisation achieved only when combined with the 8-fold dilution. These results establish that while chromatographic separation significantly reduces the MCP concentration, subsequent dilution remains essential for complete neutralisation in disinfectant validation studies.

### 3.4. RG-4 Agent Validation Confirms Concentration-Dependent EBOV Inactivation

In accordance with the 2023 National Disease Control and Prevention Agency’s testing standards (requiring ≥4.00 log_10_ TCID_50_ reduction with positive controls ≥ 5.00 log_10_ TCID_50_/mL), we evaluated the virucidal efficacy of two MCP batches (D07D1 and D08D1) against EBOV (initial titer 7.3 log_10_ TCID_50_/mL) [20]. Viral suspensions containing 6.0 log_10_ TCID_50_ EBOV were treated with varying concentrations of MCP (1%, 2.5%, and 5%) for different exposure times. Following treatment, the samples were processed through size-exclusion chromatography (MicroSpin S-400 HR columns), and the eluates were diluted 8-fold in assay medium before being inoculated onto Vero E6 cell monolayers.

To rigorously assess viral inactivation, all samples underwent three sequential blind passages with concurrent monitoring for cytopathic effects and qRT-PCR analysis. As shown in Table 3, the results demonstrated concentration- and time-dependent virucidal activity, with complete inactivation of 6 log_10_ TCID_50_ EBOV achieved within (i) 5 min with 1% MCP, (ii) 2 min with 2.5% MCP, and (iii) 1 min with 5% MCP. The appropriate positive controls consistently maintained the expected viral infectivity throughout all the experimental procedures, confirming the validity of the inactivation results.

This comprehensive evaluation revealed that both MCP batches meet and exceed the stringent requirements for high-level disinfection in BSL-4 facilities. The combination of rapid virucidal activity and reliable neutralisation protocols establishes MCP as an effective disinfectant for EBOV decontamination applications.

## 4. Discussion

This study systematically evaluated three neutralisation methods for the dual-quaternary ammonium compound disinfectant Micro-Chem Plus™ (MCP), establishing an optimised validation framework for high-containment laboratories. Our findings demonstrate that a 400-fold dilution effectively eliminates cytotoxicity but requires high viral titers (≥6 log_10_ TCID_50_/mL), whereas chromatographic separation coupled with supplemental dilution provides the most reliable neutralisation despite greater operational complexity. Validation using Ebola virus confirmed the potent virucidal activity of MCP, achieving a ≥6 log_10_ TCID_50_ reduction.

Each method has distinct advantages and limitations. Dilution is simple and consistent but reduces detection sensitivity; chemical neutralisation introduces cytotoxicity and requires extended incubation; chromatography achieves complete removal of disinfectant but is associated with increased costs and biosafety risks [21,22]. These trade-offs underscore the importance of selecting methods based on specific experimental and operational needs.

Several challenges remain. Current approaches lack standardised protocols for novel disinfectant formulations, and rapid kinetic assessment (e.g., within 1–2 min of exposure) remains problematic. Future work should prioritise the development of broad-spectrum neutralisers, harmonising validation standards across biosafety levels, and the incorporation of advanced detection technologies [23,24]. Furthermore, continuous evaluation against emerging viral threats is essential to sustain robust biosafety preparedness. Such advancements will improve accurate efficacy assessments against diverse pathogens and contribute to global health security.

This work provides a comprehensive and practical framework for validating disinfectant neutralisation methods at high-containment facilities. Our results enhance the understanding of dual-QAC disinfectant validation and offer actionable guidance for laboratory operations. As microbial threats continue to evolve, further refinement of these strategies will be critical to ensure reliable disinfection and maintain effective biosafety protocols.

## Figures and Tables

**Table 1:** Experimental design for assessing the cytotoxicity of neutralising.

Group	Treatment	Purpose
A	Neutralising agent only	Baseline cytotoxicity
B	5% MCP + Neutralising agent	Neutralisation efficacy
C	5% MCP alone	Disinfectant toxicity
D	Assay medium (DMEM + 2% FBS)	Reference viability

**Table 2:** Experimental design for evaluating virucidal activity and neutralisation efficacy.

Group	Treatment	Purpose
I	5% MCP + VSV-GFP, 5 min	Determine disinfectant virucidal efficacy
II	[5% MCP + VSV-GFP] → Neutralised	Assess neutralisation process efficiency
III	Neutraliser + VSV-GFP	Exclude direct viral inhibition by neutralisers
IV	[5% MCP + Neutraliser] + VSV-GFP	Detect residual disinfectant activity and potential assay interference
V	Untreated VSV-GFP	Confirm baseline viral infectivity
VI	Assay medium only	Establish assay baseline

**Table 3:** Efficacy of MCP against EBOV in the suspension test.

	Percentage of Disinfectant							
	0		1		2.5		5	
Treatment Time, min	D07D1	D08D1	D07D1	D08D1	D07D1	D08D1	D07D1	D08D1
1	+	+	+	+	+	+	−	−
2	+	+	+	+	−	−	−	−
5	+	+	−	−	−	−	−	−

+, Positive for viral replication, with evident cytopathic effects and detectable genomic RNA through serial passages. −, Negative for viral replication, showing neither cytopathic effects nor detectable genomic RNA (<10 copies/μL) in the third passage.

## Data Availability

Data is contained within the article.
